# Research trends and hotspots of circulating tumor DNA in colorectal cancer: a bibliometric study

**DOI:** 10.3389/fonc.2025.1492880

**Published:** 2025-05-14

**Authors:** Lele Zhang, Yuzhe Zhang, Lei Chen, Xu Wang, Yulian Liu, Yishan Huang, Yu Song, Ye Zhang, Jiandong Tai

**Affiliations:** ^1^ Department of Colorectal & Anal Surgery, General Surgery Center, The First Hospital of Jilin University, Changchun, China; ^2^ The First Laboratory of Cancer Institute, The First Hospital of China Medical University, Shenyang, China; ^3^ Department of Traditional Chinese Medicine, Chongqing Hospital of Jiangsu Province Hospital, Chongqing, China

**Keywords:** colorectal cancer, circulating tumor DNA, bibliometric, treatment, MRD

## Abstract

**Introduction:**

Colorectal cancer (CRC) is the third most common cancer worldwide and the second leading cause of cancer-related deaths. The current standard of care for patients with early-stage CRC includes surgical resection and, in selected patients, adjuvant chemotherapy. Circulating tumor DNA (ctDNA) testing is an important component of liquid biopsy, and with the development of testing technology, its value for clinical application has attracted widespread attention. The aim of this study was to help researchers review what has been achieved and better understand the direction of future research through bibliometric analysis.

**Methods:**

We used the Web of Science Core Collection database to search for ctDNA in CRC-related articles published between 2014 - 2023. Bibliometric analyses of major keywords, authors, countries, institutions, literature and journals in the field were performed using CiteSpace and VOSviewer.

**Results:**

The number of publications in the field has continued to increase over the last decade. The United States has the highest number of publications, and Italian research scholars have made outstanding contributions. Cancers is the journal with the highest number of publications.

**Conclusion:**

This study systematically summarizes the research findings in the field of ctDNA in CRC from 2014 to 2023 and describes the research hotspots and trends worldwide that can guide future research.

## 1 Introduction

Colorectal cancer (CRC) has the third highest incidence and second highest mortality rate in the 2022 global cancer statistics, with serious implications for families and societies (1). And in many high-income countries, the prevalence among people under 50 is on the rise, with rates increasing by 1-4 percent per year (1, 2). There are regional differences in 5-year survival for CRC. In developed countries, 5-year survival for all stages of colorectal cancer can exceed 60 percent; however, in developing countries, this figure drops to about 40 percent (3). Metastases are detected in approximately 25 percent of CRC patients at the time of diagnosis and occur in another 25-35 percent of patients during the course of their disease (4). Of patients diagnosed with metastatic colorectal cancer (mCRC), approximately 70-75 percent survive more than 1 year, 30-35 percent more than 3 years, and less than 20 percent more than 5 years (5). The current standard treatment for patients with early stage CRC consists of surgical removal of the tumor followed by adjuvant chemotherapy in selected patients (6, 7). Patients with rectal cancer may need to receive neoadjuvant (chemo-)-radiotherapy or total neoadjuvant treatments before surgery, depending on the tumor condition, to shrink the tumor and increase the success rate of the surgery, and some patients may receive adjuvant chemotherapy after surgery to reduce the risk of recurrence (7). Adjuvant chemotherapy improves disease-free survival (DFS) and overall survival (OS) in patients with locally resected CRC, but there is also evidence that not all patients benefit from adjuvant therapy (8, 9). As a result, many scholars are committed to developing new technological tools to assist in the diagnostic and therapeutic processes of CRC.

Liquid biopsy denotes the use of body fluid testing to obtain diagnostic information similar to that of tissue biopsy, of which circulating tumor DNA (ctDNA) testing is an important component (10). CtDNA is derived from necrotic, apoptotic tumor cells and is a component of cell-free DNA (cfDNA) (11).Compared with tissue biopsy, ctDNA testing has the advantages of easy sample access, low risk, and repeatable dynamic monitoring (12).However, due to the highly fragmented, low abundance, and low stability properties of ctDNA, this is a demanding assay that requires a high degree of sensitivity (13). Also, the specificity of ctDNA testing is critical, as processes such as clonal hematopoiesis can trigger false positives, and researchers are working to develop new techniques to more accurately distinguish between tumor and non-tumor DNA. With the development of digital polymerase chain reaction (ddPCR) and next-generation sequencing (NGS) technologies, which have significantly improved the accuracy of ctDNA detection, a large number of clinical studies focusing on the value of ctDNA for clinical applications have attracted widespread attention.

The promising role played by ctDNA in the diagnosis and treatment of patients with CRC has been supported by an increasing number of studies (14). For patients with mCRC, ctDNA can be adopted in certain circumstances to drive targeted therapeutic strategies, as supported by several clinical trials but also as increasingly endorsed by medical oncologists in their daily practice. Besides, in the metastatic setting, ctDNA could be soon employed to assess therapeutic efficacy and guide the adjustment of treatment regimens (15). In the adjuvant/neoadjuvant setting, ctDNA can be detected in patients’ postoperative blood and clinical trials are now assessing the clinical utility of ctDNA to predict and monitor the risk of recurrence, and provide personalized adjuvant therapeutic recommendations for patients (16, 17). Currently, several ctDNA clinical trials for mCRC are underway, and with the continuous advancement of ctDNA detection technology and in-depth clinical research, the prospect will be even broader (18).

Bibliometrics first appeared at the turn of the century, became an independent discipline in 1969, and is widely used for literature analysis (19). Bibliometrics can be used to summarize progress on a research topic, identifying hotspots or emerging trends and contributions from authors, journals, institutions, or countries. Although there have been several previous reviews on the use of ctDNA in CRC, the focus has been different, and there is still a lack of a comprehensive and visual analysis of the field from a bibliometric perspective. In this study, we used a bibliometric approach to summarize relevant studies in the field from 2014-2024, assessing the literature published in different countries, authors, institutions, and journals. We also systematically analyzed important research directions in the field, highlighting research findings of significance and pointing out future research directions.

## 2 Materials and methods

**Figure 1:**
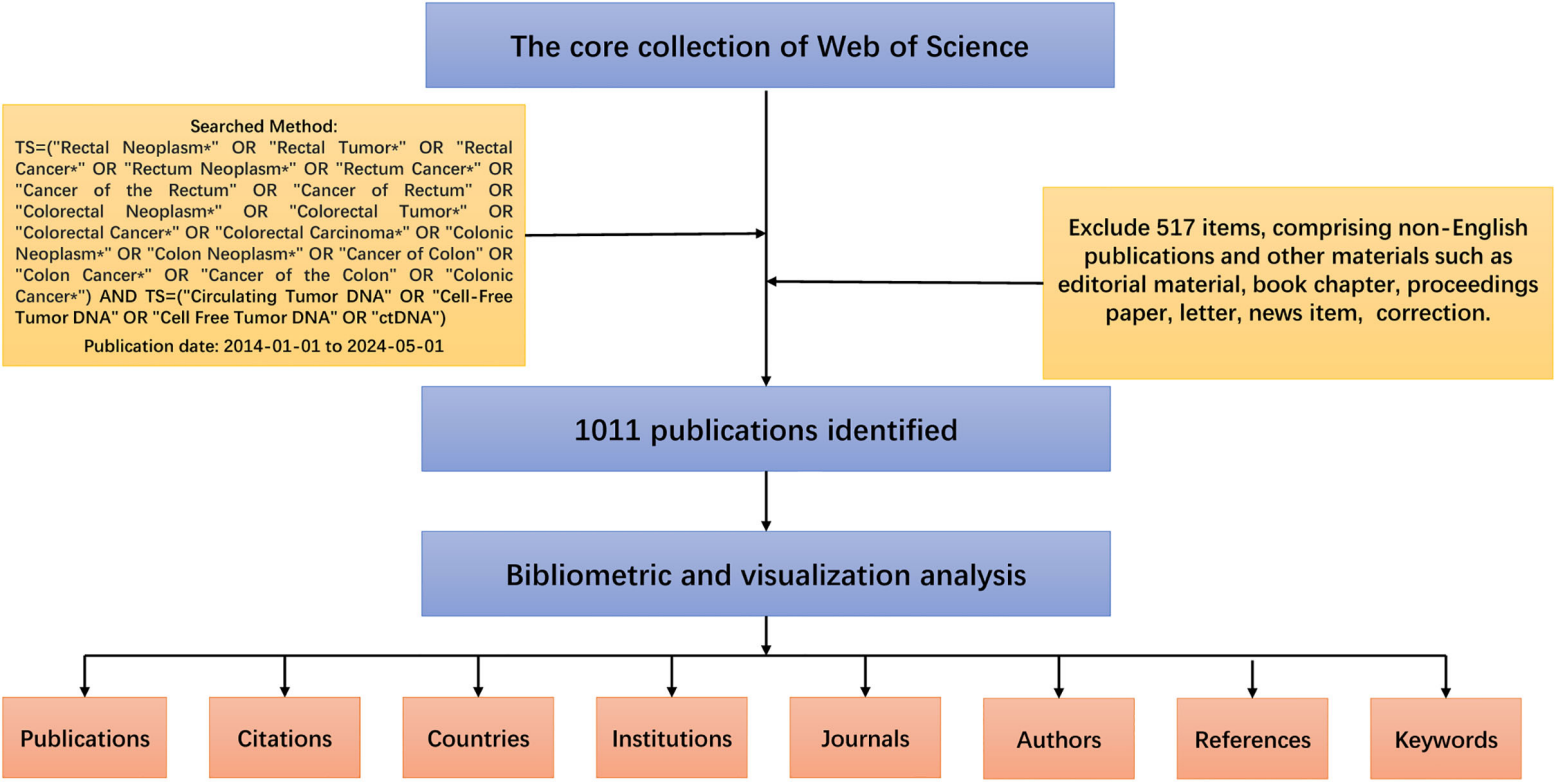
Detailed flowchart steps of the search strategy in screening publications.

In this study, the Web of Science Core Collection (WoSCC) was chosen as the database for bibliometric analysis. This database is a feature-rich database of high-quality digital literature resources that not only has sufficient bibliometric indicators but also possesses a strict screening mechanism (20, 21). It includes only important academic journals and important international academic conferences in various subject areas and is selected in a neutral and unbiased manner. It also includes references cited in the literature for our subsequent in-depth analyses. Our search strategy and exclusion criteria are shown in **Figure 1**, and a total of 1011 documents were obtained for subsequent bibliometric analyses. This strategy, whilst useful for collecting a wide range of information, may include literature that is not specific to CRC, for which a rigorous initial screening was carried out, with two researchers independently checking and manually excluding publications that were not relevant to the topic to ensure that the included literature was, at the very least, relevant to CRC. All data retrieval was completed within one day to minimize bias due to database updates. Finally, results were validated using CiteSpace to ensure there were no duplicates.

Title, authors, year of publication, country/region, institution, keywords, citations, abstracts, and references are from the WOSCC database. The downloaded files were in plain text format. All analyses were completed on May 5, 2024, to exclude the effect of database updates on the study results. The visualization tools VOSviewer (v.1.6.19), CiteSpace (6.2.R4), and Scimago Graphica (v.1.0.36) were used in this study (22–24). VOSviewer and CiteSpace are classic bibliometric analyzers that show the current status and research trends in a particular research area through beautiful visualizations such as authors, countries, institutions, and co-cited keywords. In addition, the number of articles published in each country in the field of ctDNA in CRC was visualized using Scimago Graphica.

## 3 Results

### 3.1 Analysis of annual publication trends

**Figure 2:**
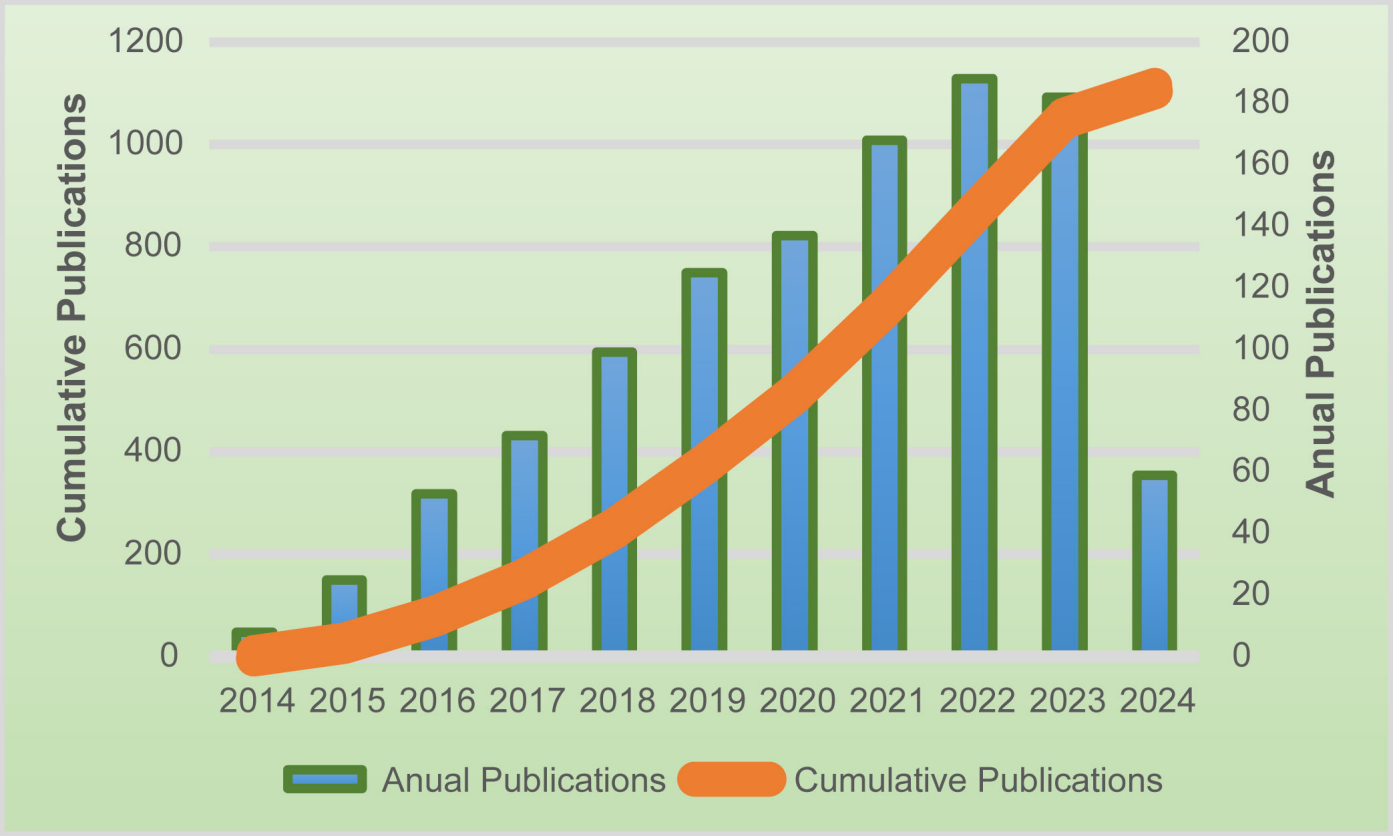
The number of publications in the last ten years, as well as the trend of annual publication increase.

From 2014 to 2024, a total of 1011 articles were eligible for the search. The number of published articles on ctDNA in CRC is shown in **Figure 2**. We can see that the number of articles published in this field continues to grow each year. From 2019 onwards, the number of annual publications stays above 100 and reaches a peak in 2022 (188 papers). Between 2021 and 2023, the number of papers remains relatively stable at a high level.

### 3.2 Analysis of the contributions of countries

**Figure 3:**
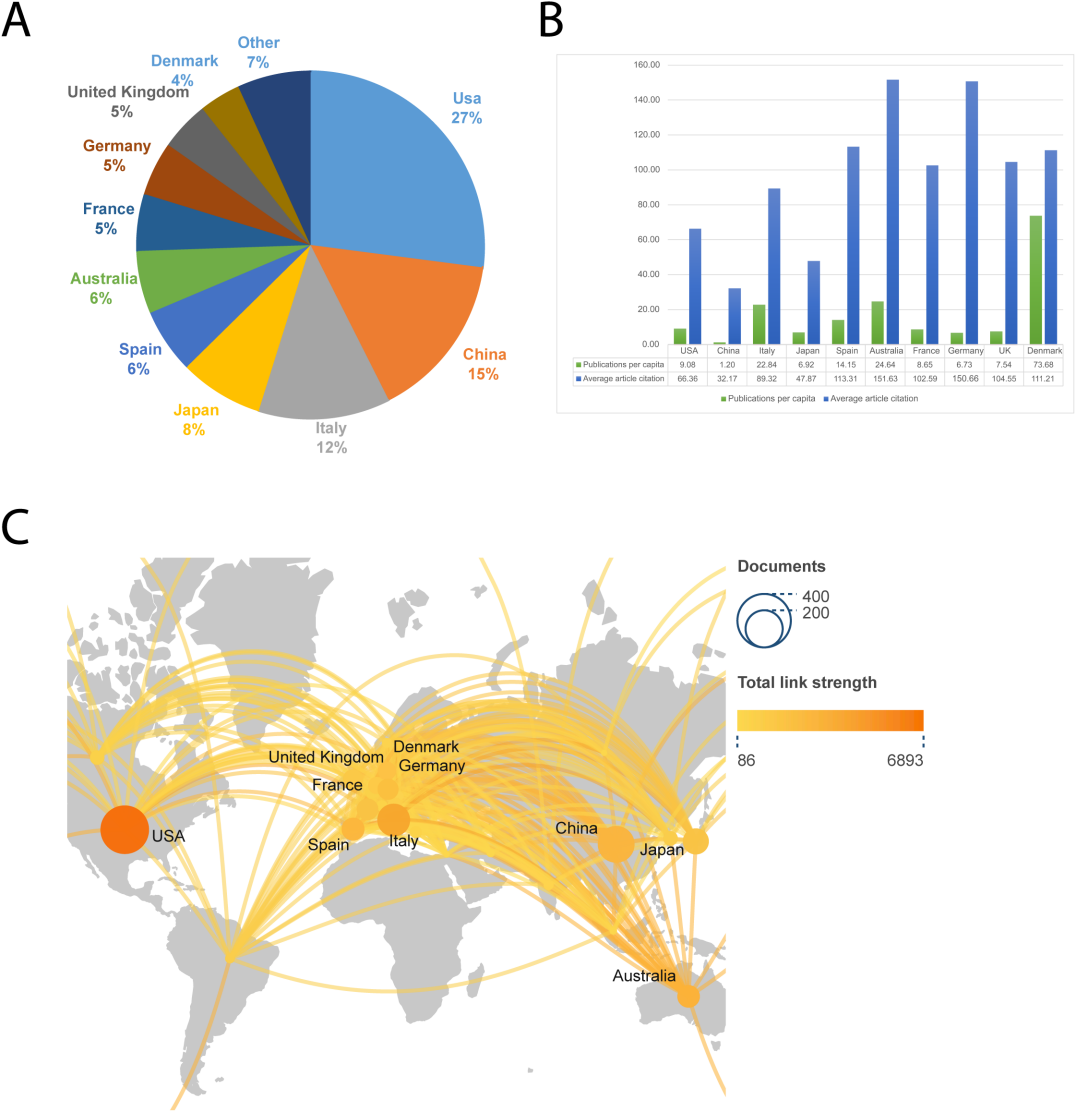
The ten countries with the most publications. **(A)** Percentage of the ten countries with the highest number of publications. **(B)** The top ten countries’ number of publications per capita and average article citations. **(C)** A global map depicting the number of publications by country. The shade of the circle represents the number of publications, while the thickness of the line represents the intensity of collaboration between the two countries.

The United States published the largest number of articles, 302, or 27 percent of the total number of articles. The ten countries with the highest number of articles each as a percentage of the total number of articles are shown in **Figure 3A**, with the United States, China, and Italy in the top three. We have normalized the number of publications by the population of each country in **Figure 3B** to take into account the effect of the population size factor, and we have also added the average number of citations to the articles. We can see that Denmark has the highest number of publications per capita in this field, with Australia and Germany having higher average article citations. **Figure 3C** shows the collaborative network of countries involved in this research area, and it can be observed that European countries (including Italy, Spain, France, Germany, the United Kingdom, etc.) are more interested in this research area.

### 3.3 Analysis of institutions

**Figure 4:**
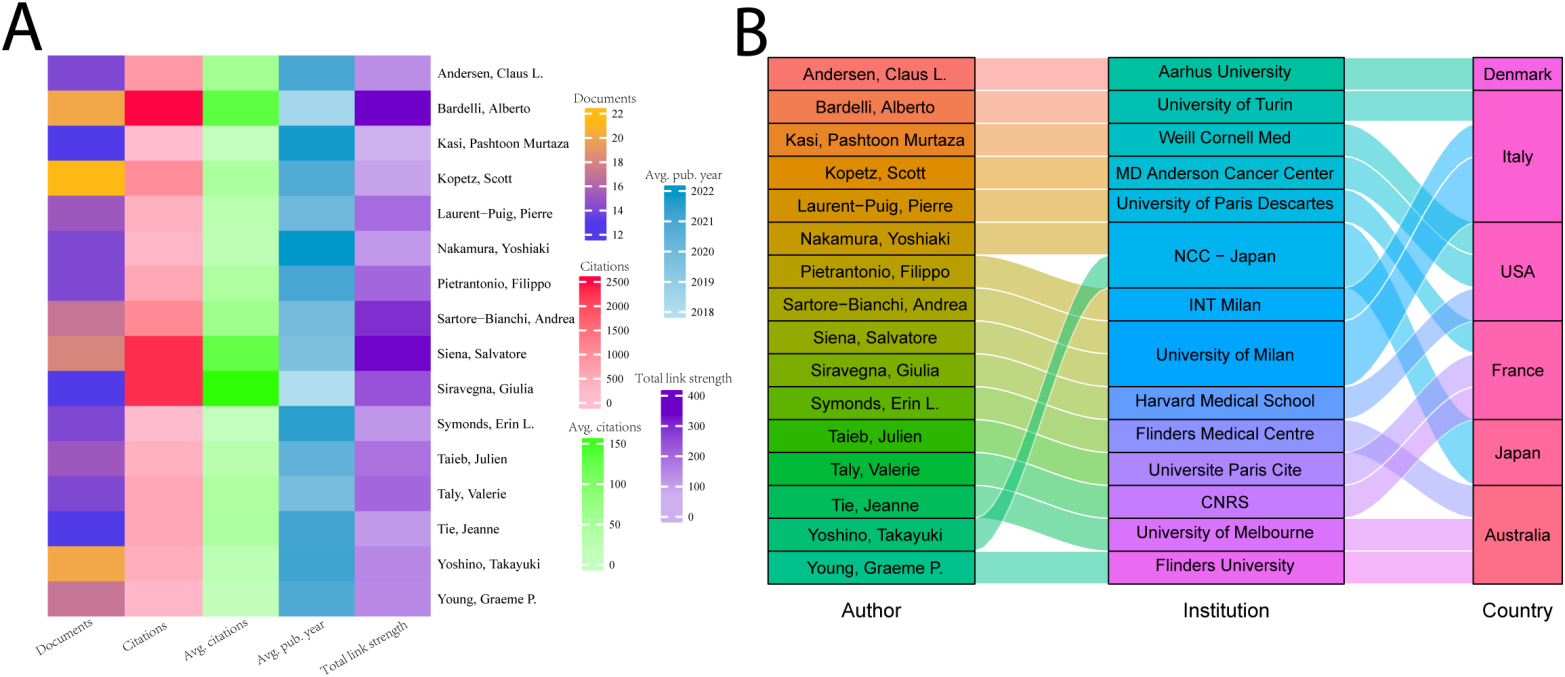
The ten institutions with the most publications. **(A)** Map of the top fifty-seven most productive institutions’ collaboration networks. **(B)** The top ten institutions’ number of publications and average number of citations.

When the minimum number of articles published by an institution was set at 10, a total of 58 institutions met the criteria. These 180 productive papers were analyzed for co-authorship using VOSviewer. **Figure 4A** depicts the collaborative network of research institutions. These institutions form clusters of collaborative networks, indicated by various colors. Based on the number of publications, **Figure 4B** shows the top 11 institutions, with the University of Texas MD Anderson Cancer Center having the most publications (47), followed by the National Cancer Center Hospital East (32). According to our data, the University of Turin has the highest number of citations (6591) as well as the average number of citations (287), suggesting that the institution produces a large amount of influential research.

### 3.4 Analysis of authors

**Figure 5:**
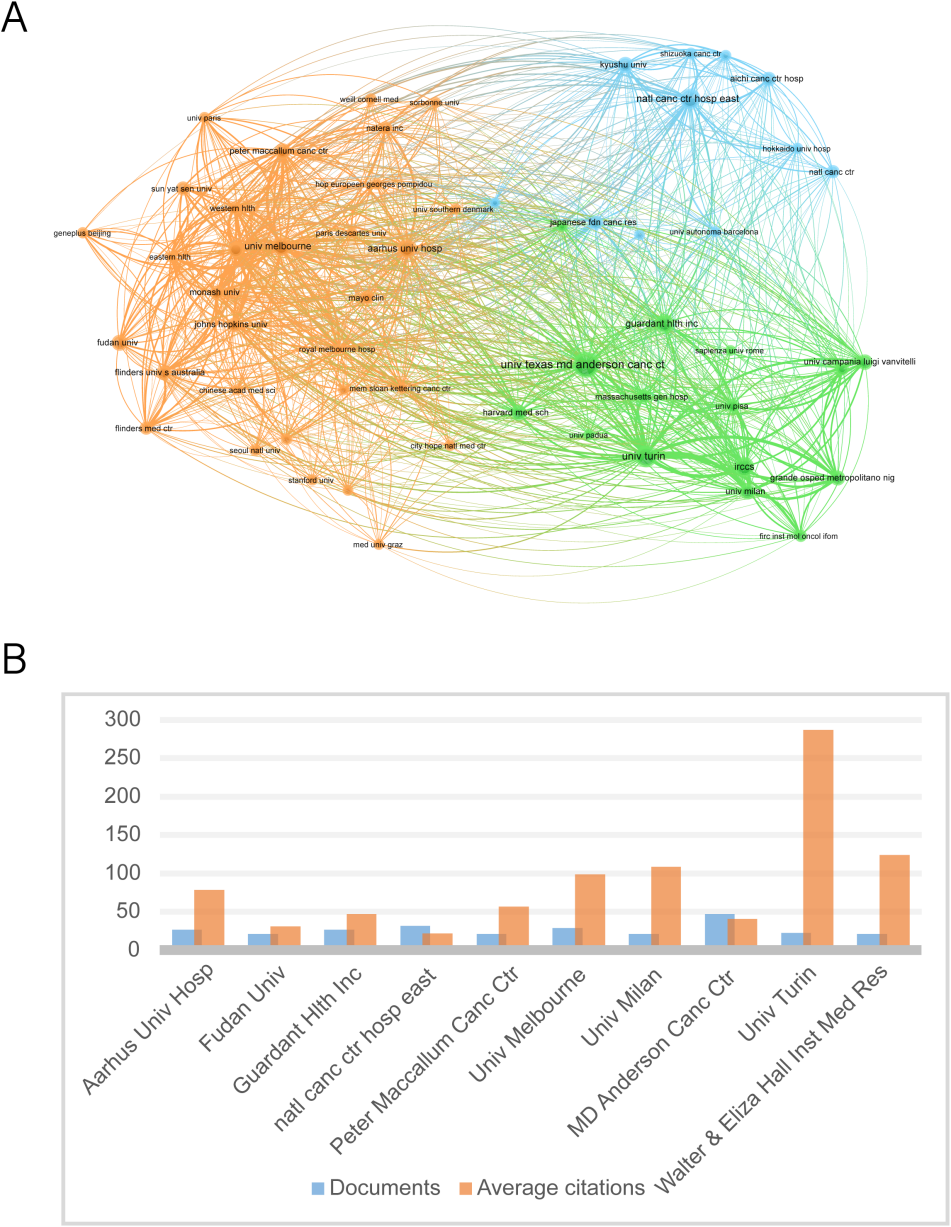
The top 16 productive authors. **(A)** The top 16 authors’ number of papers, total citations, average number of citations, intensity of collaboration, and average time to publication. **(B)** Institutional affiliation and country of the top 16 authors with the most publications.

Correlation analyses of authors help to identify core authors and major collaborations in the field. We analyzed the top 16 authors in terms of number of publications who have published more than 13 papers in the field. As shown in **Figure 5A**, Scott Kopetz published the largest number of publications (22) in the field; Alberto Bardelli has the most citations (2,611), as well as the strongest collaborations. Giulia Siravegna has the highest average number of citations (about 175). Yoshiaki Nakamura’s study’s average publication date is 2022, closest to the present. In **Figure 5B**, we show the relationship between these 16 authors, their countries, and their affiliations. Four of these 16 researchers were from Italy, and three each from the United States, France, and Australia. The top 5 highly cited authors are all from Italy and the US.

### 3.5 Analysis of the contributions of journals

**Figure 6:**
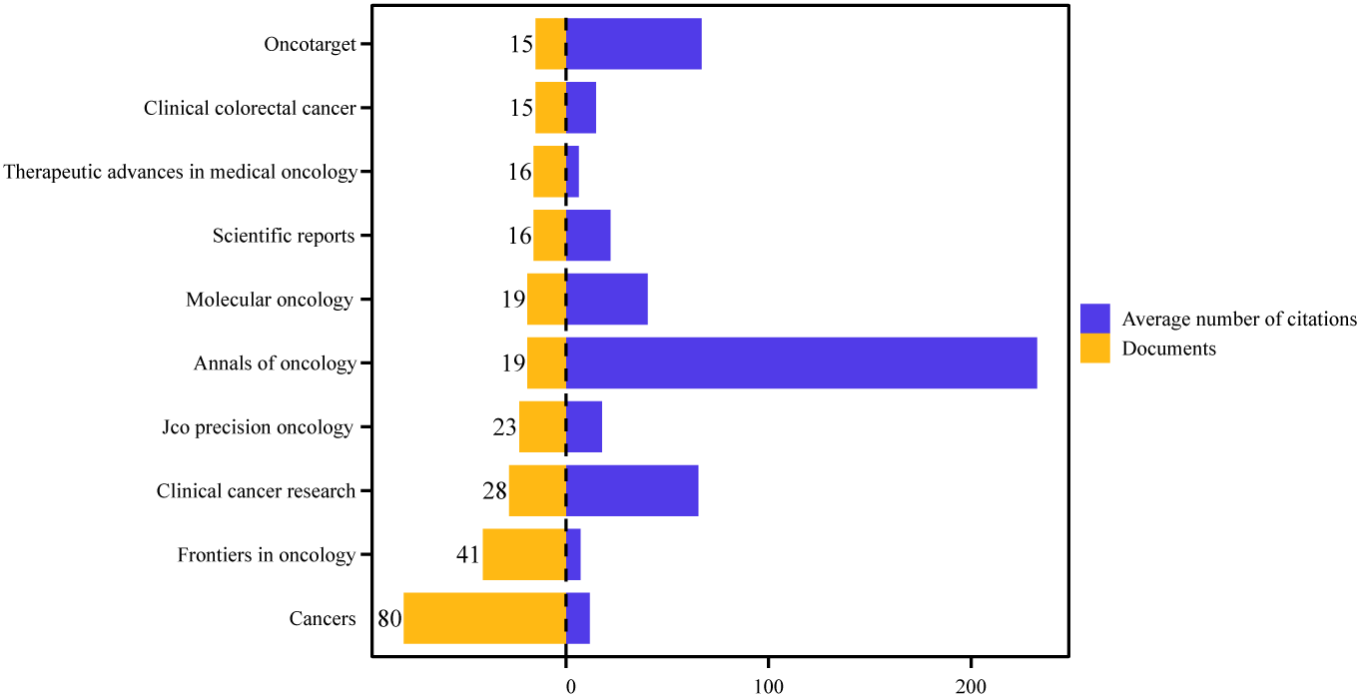
The number of publications and the average number of citations for the top ten journals.

**Figure 6** illustrates the ten journals with the highest number of publications in the field. Based on our data, these ten journals published 26.9% (272/1011) of the total number of publications. *Cancers* was ranked first with 80 articles and had the strongest collaboration strength. *Annals of Oncology* had the highest number of citations (4418) as well as the highest average number of citations (233), which demonstrates the high impact of the relevant literature published in this journal. Among the top 10 journals, most were JCR Q1 and Q2.

### 3.6 Analysis of highly cited references

The top 10 most cited articles in the field are shown in **Table 1**, and this literature is an important reference for scholars engaged in this research. The citation counts of the top 10 most cited articles ranged from 723 to 3612. The article titled *Detection of circulating tumor DNA in early- and late-stage human malignancies* was the most cited article with 3612 citations. In addition, two of the ten articles were published by Giulia Siravegna, demonstrating her outstanding contribution to this field, and three were published in the same journal called *SCIENCE TRANSLATIONAL MEDICINE.*

**Table 1:** The ten most cited articles.

Rank	First Author	Year	Journal Title	Source	Citations
1	Bettegowda, C (25)	2014	Detection of circulating tumor DNA in early- and late-stage human malignancies	SCIENCE TRANSLATIONAL MEDICINE	3612
2	Van Cutsem, E (26)	2016	ESMO consensus guidelines for the management of patients with metastatic colorectal cancer	ANNALS OF ONCOLOGY	2445
3	Cohen, JD (27)	2018	Detection and localization of surgically resectable cancers with a multi-analyte blood test	SCIENCE	2028
4	Wan, JCM (28)	2017	Liquid biopsies come of age: towards implementation of circulating tumour DNA	NATURE REVIEWS CANCER	1724
5	Siravegna, G (29)	2017	Integrating liquid biopsies into the management of cancer	NATURE REVIEWS CLINICAL ONCOLOGY	1334
6	Alix-Panabières, C (30)	2016	Clinical Applications of Circulating Tumor Cells and Circulating Tumor DNA as Liquid Biopsy	CANCER DISCOVERY	1047
7	Tie, J (31)	2016	Circulating tumor DNA analysis detects minimal residual disease and predicts recurrence in patients with stage II colon cancer	SCIENCE TRANSLATIONAL MEDICINE	1046
8	Phallen, J (32)	2017	Direct detection of early-stage cancers using circulating tumor DNA	SCIENCE TRANSLATIONAL MEDICINE	795
9	Cristiano, S (33)	2019	Genome-wide cell-free DNA fragmentation in patients with cancer	NATURE	723
10	Siravegna, G (34)	2015	Clonal evolution and resistance to EGFR blockade in the blood of colorectal cancer patients	NATURE MEDICINE	723

### 3.7 Analysis of keywords

**Figure 7:**
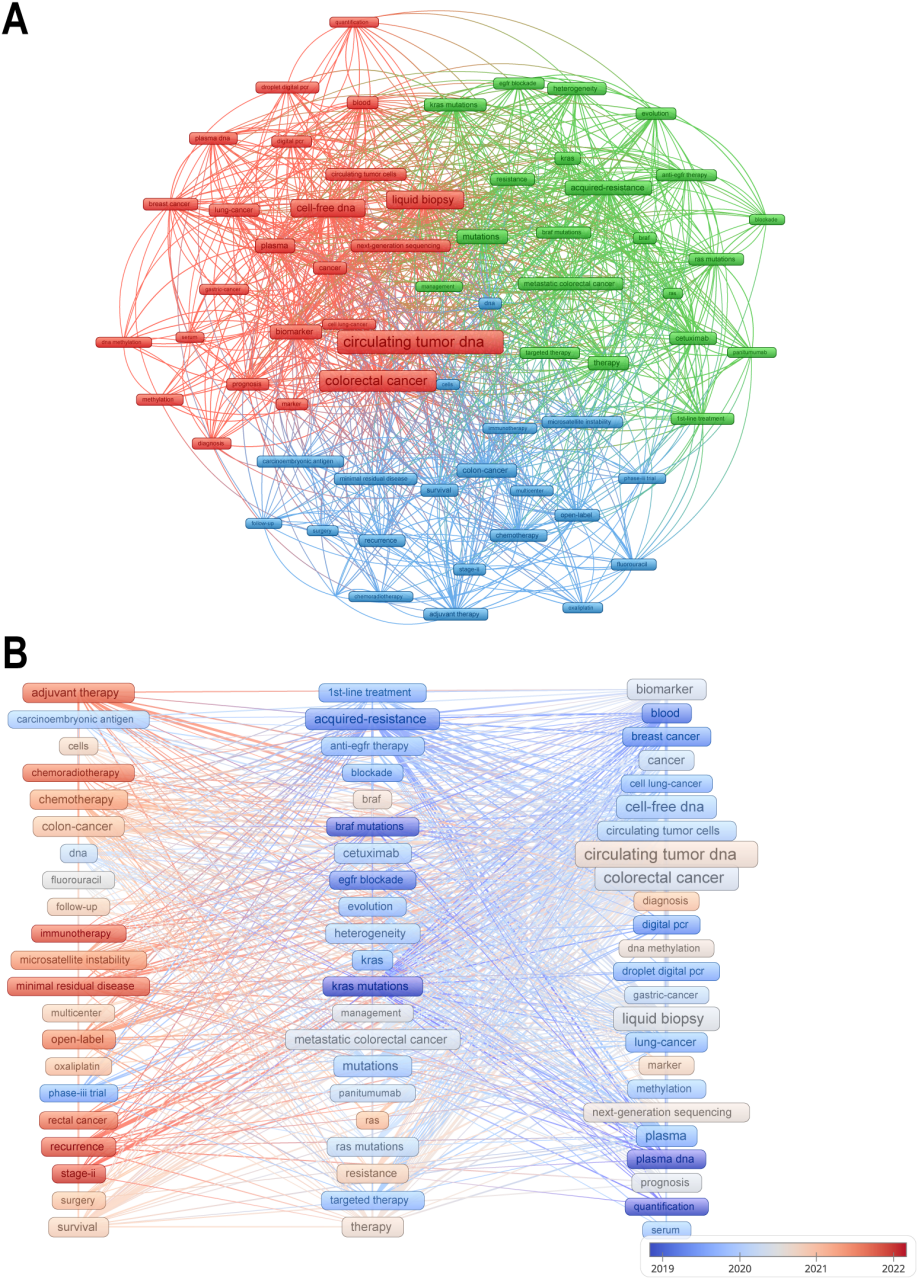
**(A)** Mapping the co-occurrence of the top 66 keywords. **(B)** A keyword’s color represents the average publication time of articles containing that keyword, with warmer hues signifying a publication time closer to the present.

Keywords cover the main topics of publications, so high-frequency keywords are well suited for co-occurrence analysis. Cluster analysis of keywords can help us understand the main research directions and future research hotspots in the field. We set the minimum number of keyword occurrences to 30, obtained 66 keywords, and divided them into three clusters. **Figure 7A** shows the visual network map of these 66 keywords in the three clusters and their co-occurrences, with three different colors to indicate the different clusters, and items with the same color in the same cluster imply a higher correlation with each other. Node labels indicate keywords and the size of each node indicates the frequency of occurrence of the keyword. A link connecting two nodes indicates a co-occurrence relationship between two keywords. The red cluster contains 24 keywords, mainly including circulating tumor DNA, colorectal cancer, liquid biopsy, etc.; the green cluster contains 21 keywords, including acquired-resistance, mutations, metastatic colorectal cancer, etc.; the blue cluster also contains 21 keywords, including adjuvant therapy, survival, recurrence, etc. The keywords circulating tumor DNA, colorectal cancer are located in the center of the visualized network graphs.

In order to explore evolutionary trends over time, keywords are colored in **Figure 7B** according to the average time of publication. Cooler colored keywords represent earlier appearing keywords and warmer colored keywords represent later appearing keywords. The most recently appearing keywords are adjuvant therapy, immunotherapy, and recurrence. Chemotherapy- and immunotherapy-related studies have gradually gained importance in recent years, and these studies are often closely aligned with prognostic analyses, which is also consistent with the results of our cluster analysis.

### 3.8 Analysis of the main content of the articles

By analyzing the articles we retrieved, we found that 35, 107, and 177 articles focused on early-detection and screening, adjuvant/neoadjuvant therapy, and metastatic colorectal cancer (mCRC), respectively. This fully demonstrates that the application of ctDNA can comprehensively cover the diagnosis and treatment process of CRC. At the same time, it also reveals that the main focus of global clinical research is on the treatment of CRC, especially mCRC, which suggests that we need to increase the attention and investment in early detection and screening of CRC.

## 4 Discussion

This study comprehensively analyses the research trends, research hotspots and future prospects of global ctDNA application in CRC over the past decade using the classic bibliometric software VOSviewer and CiteSpace, in order to help academics better understand the current status of research in this field, reduce the blindness of research topic selection, and provide researchers with new perspectives and directions to promote the application of this field in clinical practice.

### 4.1 Trends in publications: technology development and clinical applications

Annual publication volume exceeds 100 after 2019 (peaks at 188 in 2022), a growth trajectory that highly overlaps with the maturity of ctDNA detection technologies (e.g., upgrade of ddPCR to NGS platforms) and the explosion of clinical validation studies (e.g., pivotal trials, such as IDEA 2021, DYNAMIC 2022, etc.) (35–40). Notably, the number of publications has stabilized at more than 150 in 2021-2023, suggesting that the field has transitioned from the early stage of technology exploration to clinical validation and guideline integration. For example, the American Society of Clinical Oncology (ASCO) and the European Society For Medical Oncology (ESMO) affirmed the important role of ctDNA in the expert guidelines on CRC published in 2022, which is directly contributing to a surge in translational clinical research on ctDNA (41–43). However, an analysis of the content of the published papers exposed an imbalance in the direction of research in this field, with the majority of papers focusing on the area of prognostic assessment, while research on the application of ctDNA in the dynamic monitoring of treatment and early screening is still insufficient, which may be a key breakthrough point for the clinicalization of the technology in the next phase.

### 4.2 National contribution: geo-research ecology

The high output volume of papers issued in the US (302, 27%) reflects its resource bias towards translational research in emerging technologies; however, Denmark’s leadership in per capita paper volume suggests that localized regions can develop academic impact by pinpointing niche areas. The high citation frequency of Germany and Australia may be closely related to their focus on multicenter clinical trial design and interdisciplinary collaboration (40, 44–46). It is worth noting that the close collaborative network between European countries may have accelerated the efficiency of the translation of ctDNA technology from the laboratory to EU CE marking, a model of regional synergistic innovation that is worthy of emulation in other regions (47).

### 4.3 Institutional co-operation: knowledge production networks and clinical translation

Top institutions such as MD Anderson Cancer Center (most publications) and the University of Turin (most citations and average citations) represent two innovation pathways: the former validates the prognostic value of ctDNA through large-scale clinical cohorts (e.g., the PROSPECT trial), while the latter relies on basic molecular pathology research (e.g., evolutionary tracking of KRAS clones) to drive the sensitivity of detection technologies (48–51). VOSviewer clustering analysis shows that the institutional collaboration network is characterized by the triple node of ‘clinical center - molecular diagnostic company - regulatory agency’ (**Figure 4A**). For example, the joint study between Guardant Health and Mayo Clinic directly led to the accelerated approval of Guardant360 CDx by the FDA and led to important publications in the field (52, 53). This industry-academia-research synergy model will significantly shorten the time cycle of ctDNA technology from discovery to clinical application, but it may also lead to potential conflicts between commercial interests and academic independence, and a more transparent mechanism for disclosing interests needs to be established in subsequent research.

### 4.4 Author impact: academic leaders driving clinical change

Core authors such as Alberto Bardelli (2,611 citations) have provided a theoretical cornerstone for the determination of the time window for individualized treatment through a large number of studies exploring the dynamic association between ctDNA and tumor heterogeneity, while the studies of Scott Kopetz’s team have directly rewritten the treatment guidelines of CRC (54–57). The current geographic distribution of authors is still Euro-American centric, which may limit the universal validation of ctDNA technology in the context of different ethnicities and medical resources, and there is an urgent need to include researchers from Asia, Africa and Latin America to enhance the external validity of the findings.

### 4.5 Journal ecology: barriers to knowledge integration

Although *Cancers* is the most active knowledge dissemination platform with 80 publications, its low average citation frequency contrasts sharply with *Annals of Oncology’s* superior impact (233 citations), and this ‘quantity-quality’ divide exposes a structural contradiction in the knowledge chain in the field: the former, as an open access journal, promotes methodological primitivism through rapid publication, whereas the latter, with its high guideline citation rate, is the preferred outlet for phase III randomized controlled trials (47, 58). At the same time, there is a significant difference in the publication journals of basic research articles and large clinical trial articles, with the latter mostly published in Q1 journals, which makes it more difficult to incorporate evidence of molecular mechanisms into guideline updates. It is recommended to promote the establishment of a fast connection channel between academic paper pre-publication platforms and academic journals, so that innovations at the laboratory stage can enter the clinical validation process more quickly.

### 4.6 Highly co-cited literature: anchors and limits of academic consensus

Highly co-cited literature constitutes the cornerstone work in the field, and its impact is reflected in three aspects: methodological innovation, clinical validation paradigm establishment, and research direction guidance. First, Bettegowda et al. (2014, 3612 citations) systematically demonstrated for the first time the broad applicability of ctDNA in advanced solid tumors, a finding that not only established the potential of ctDNA as a pan-cancer marker, but also pushed subsequent studies to focus on improving the sensitivity of the technology (25, 32). Secondly, two highly cited articles from Siravegna’s team revealed the unique value of ctDNA in monitoring treatment resistance, driving the shift of ctDNA from a prognostic tool to a real-time treatment guidance tool (29, 34). However, whereas earlier studies have focused on patients with advanced disease, the prospective cohort study by Tie et al. demonstrated that patients at high risk of recurrence after surgery for stage II colon cancer can be identified by testing ctDNA, which can help guide adjuvant therapy (59). Finally, while CancerSEEK’s multi-omics model enables screening for eight early cancers, its sensitivity in breast cancer is only 33% and its cost-effectiveness is unproven, forcing the field to turn to low-cost dimensions such as fragmentomics (27, 33). Current highly cited studies are mostly based on European and American cohorts, and differences between Asian and African populations have not been systematically investigated; and there is a lack of isolated studies on early screening and postoperative surveillance, and a lack of dynamic and continuous studies that combine screening and surveillance, which are directions for future research.

### 4.7 Keyword evolution: technology-mechanism-clinical synergy

Bibliometric analyses in this study revealed three distinct keyword clusters, each representing a key dimension of current scientific enquiry: the technology development validation layer (red cluster), the mechanism analysis exploration layer (green cluster), and the clinical therapeutic decision layer (blue cluster). Together, these three areas paint a three-dimensional picture of ctDNA development in colorectal cancer research.

The red clusters are centered on ‘ctDNA’ and ‘liquid biopsy’, and are strongly correlated with technical indicators such as ddPCR and NGS, reflecting the development of testing technologies in this field and highlighting the centrality of technology iteration. The term liquid biopsy was coined about a decade ago to refer to the use of body fluids as a means of obtaining the same diagnostic detail as tissue biopsy, of which ctDNA testing is a key component (10). Liquid biopsies have many advantages over traditional biopsies, including easy access, low risk to the patient, and dynamic observation (12). However, there are still many challenges in the current application of ctDNA, for example, ctDNA is highly fragmented, low abundance and low stability, which requires high sensitivity of detection methods (13). The first high-throughput digital polymerase chain reaction method, known as BEAMING, is currently used in a wide range of applications, such as detecting KRAS mutations in the plasma circulation of colon cancer patients, and it has also been extended for the detection of methylated fragments (60–62). In contrast, non-targeted approaches that do not rely on *a priori* knowledge using next-generation sequencing (NGS)-based methods have the ability to detect a much larger number of variants in multiple genes, aiming for a comprehensive analysis of the tumor genome (37). The use of CtDNA in colorectal cancer is inextricably linked to the development of its detection technology (63). However, there are many methods available for analyzing ctDNA and there is an urgent need for consistent SOPs and standardized methods, taking into account all possible analytical and pre-analytical factors that may affect test results (64). Most ctDNA studies have focused on advanced cancers with relatively high ctDNA concentrations, and there is a lack of detailed experience with early cancers and low ctDNA concentrations.

The green cluster represents the mechanism analysis exploration layer, and the co-occurrence of the keywords ‘acquired-resistance’ and ‘KRAS mutations’ reflects the focus on overcoming drug resistance. The emergence of drug resistance during adjuvant therapy is a common clinical problem, and genetic mutations are an important reason for patients to develop drug resistance. KRAS wild-type colorectal tumors are usually sensitive to EGFR blockade, e.g., cetuximab and panitumumab are one of the mainstays of targeted therapy for colorectal cancer, but resistance almost always develops within a few months of initiating therapy. that the emergence of KRAS mutations is a mediator of acquired resistance to EGFR blockade (61, 65). We can use ctDNA to monitor the course of therapy and thus guide anti-EGFR rechallenge therapy. the molecular basis for this is that mutant KRAS alleles detected by ctDNA begin to decline, even to undetectable levels, after cessation of anti-EGFR therapy and the population recovers drug sensitivity (34). The CHRONOS trial, a prospective clinical study in which researchers used ctDNA analysis to select mCRC patients for re-initiation of panitumumab, demonstrated that genotyping of ctDNA in the blood can be safely and effectively used in the management of mCRC patients (54).

The blue clusters represent the clinical decision-making level, and the high frequency of ‘adjuvant therapy’ and ‘recurrence’ signals the migration of research to therapeutic intervention scenarios, with more and more studies investigating the use of ctDNA in the treatment of patients with colorectal cancer, providing supportive evidence for the use of ctDNA (14, 66). In the study by Reinert et al, the mean lead time from detection of ctDNA in plasma to detection of recurrence by standard computed tomography was 8.7 months (range, 0.8-16.5 months, P < 0.001) (67). The results of the DYNAMIC 2022 trial demonstrated that ctDNA-guided adjuvant chemotherapy in stage II CC patients not only reduces the use of adjuvant chemotherapy, but also leads to greater clinical benefit of adjuvant chemotherapy in ctDNA-positive patients (40).

The keyword time-series evolution in **Figure 7B** reflects to some extent the disconnect between assay technology maturity and clinical application. Early studies focused on basic explorations such as KRAS mutations and acquired resistance, while in the mid-term, they shifted to liquid biopsy technology validation and standardization, and more recently, they have focused on therapeutic scenario applications (e.g., relapse monitoring, immunotherapy). This suggests that chemotherapy and immunotherapy-related studies have gained prominence in recent years, and that these studies are often closely linked to prognostic analyses. CtDNA is able to detect key mutations in CRC patients during adjuvant therapy, assessing the response to therapy and guiding the individualization of treatment regimens accordingly to improve the prognosis of the patient (34, 68, 69). However, research at different stages is fragmented: molecular mechanisms discovered in the early stage have not been able to guide the optimization of near-term therapeutic strategies, and technological tools matured in the mid-stage have not been systematically integrated into the clinical decision-making framework. It is recommended to strengthen the synergy of research across time dimensions, to promote the closed-loop connection from basic discovery to clinical translation, and to promote the integration of multiple technologies to break the current limitations of single-dimensional analysis.

### 4.8 Advantages and prospects of multi-omics integration and AI-driven analysis in ctDNA

The application of Artificial Intelligence (AI) in the field of ctDNA has significant potential advantages and innovations, which can make up for the existing deficiencies of ctDNA and amplify its advantages (70, 71). Firstly, since ctDNA is extremely low in blood (especially in early-stage cancers) and there is a large amount of normal DNA interference. ai (e.g., deep learning, machine learning) can identify weak tumor signals by analyzing complex sequencing data, thus significantly improving detection sensitivity and specificity (72, 73). Second, AI can discover patterns from massive ctDNA data that are difficult to capture by traditional methods, such as identifying ctDNA features specific to early-stage cancer patients by training models to assist in achieving early diagnosis (74, 75). More importantly, AI-driven portable ctDNA analysis tools (e.g., based on next-generation sequencing or nanopore sequencing) can drive the development of bedside testing, extracting effective signals from low-coverage sequencing data and greatly reducing the cost of promotion (76, 77).

Multi-omics integration can significantly improve the precision of cancer detection, monitoring and treatment by combining data from different levels such as genome, epigenome, transcriptome, proteome and metabolome. Early stage of colorectal cancer has extremely low ctDNA content, and a single histological marker may lead to missed detection due to insufficient sensitivity (78–80). Multi-omics integration can significantly improve detection performance through multi-dimensional signal cross-validation (81). In addition, the use of multi-omics to mine colorectal cancer-specific combinatorial markers is currently of great interest, e.g., the combination of long-chain non-coding RNA and methylation markers in plasma can improve the detection specificity (82, 83). It is expected that the reconstruction of spatio-temporal tumor evolution maps using ctDNA and other histological data can bring new perspectives for in-depth analysis of the heterogeneity between primary and metastatic foci of colon cancer (84, 85). Clonal evolution of metastatic foci and chemotherapy resistance can be tracked through ctDNA mutations, copy number variations and methylation patterns (86, 87). Alternatively, molecular driving mechanisms of lymph node metastasis were postulated by integrating ctDNA data with primary tumor single-cell sequencing (88).

Multi-omics integration and AI-driven analysis are reshaping the boundaries of ctDNA applications: multi-omics breaks through the limitations of single biomarkers, and AI unlocks the clinical value of high-dimensional data (89). The synergy between the two will promote liquid biopsy from ‘alternative diagnostic tool’ to ‘intelligent management system for the whole tumor cycle’, and ultimately achieve the triple goals of increasing the early diagnosis rate of cancer, reversing drug resistance and fairness of medical resources (90, 91). In the future, with the accumulation of large-scale prospective trial data, these technologies are expected to become the core pillar of oncology precision medicine within ten years (92).

## 5 Limitations

As a bibliometric analysis, this study also has some limitations. All the data in this study were retrieved and downloaded from WoSCC. Although this database is the most commonly used and authoritatively comprehensive database, publications that are not included in it may be missed. The included articles may still contain non-CRC-specific articles due to limitations of the search strategy and diversity of the literature. Secondly, some important recent publications may not have received a high number of citations due to the short period of time since their publication and may not have attracted sufficient attention. Further, since we limited our study to English literature, other important studies in other languages may have been missed.

## 6 Conclusion

By reviewing publications from the past decade, this study provides a visualization of ctDNA-CRC research, revealing global trends, research hotspots and the literature base related to ctDNA in CRC. The research trajectory of ctDNA in colorectal cancer is depicted, from its origins in the development of liquid biopsy to its current role in guiding precision therapy. Together, the red, green and blue clusters highlight a translational process - from detecting early-stage tumors, to combating drug resistance, to optimizing survival. In the future, emerging technologies, particularly multi-omics integration with AI-driven analysis, promise to address long-term challenges in sensitivity and heterogeneity. However, clinical adoption depends on standardized protocols, cost reduction and evidence from large-scale trials. As ctDNA moves from biomarker to treatment decision maker, its integration into routine oncology practice will redefine colorectal cancer management, ultimately leading to an era of ‘molecular recurrence’ prevention and patient-tailored care.

## Data Availability

The datasets presented in this study can be found in online repositories. The names of the repository/repositories and accession number(s) can be found below: the Web of Science Core Collection database.
